# Oxidative stress initiates hemodynamic change in CKD-induced heart disease

**DOI:** 10.1007/s00395-024-01085-7

**Published:** 2024-10-15

**Authors:** Payel Sen, Jules Hamers, Theresa Sittig, Bachuki Shashikadze, Laura d’Ambrosio, Jan B. Stöckl, Susanne Bierschenk, Hengliang Zhang, Chiara d’Alessio, Lotte M. Zandbergen, Valerie Pauly, Sebastian Clauss, Eckhard Wolf, Andreas Dendorfer, Thomas Fröhlich, Daphne Merkus

**Affiliations:** 1 Institute for Surgical Research, Walter Brendel Center of Experimental Medicine, University Clinic Munich, LMU Munich, Marchioninistrasse 68, 81377 Munich, Germany; 2 German Center for Cardiovascular Research (DZHK), Munich Heart Alliance (MHA), Partner Site Munich, Munich, Germany; 3 Department of Medicine I, University Hospital, LMU Munich, Marchioninistrasse 15, 81377 Munich, Germany; 4 Interfaculty Center for Endocrine and Cardiovascular Disease Network Modelling and Clinical Transfer (ICONLMU), LMU Munich, Munich, Germany; 5 Gene Center, LMU Munich, Munich, Germany; 6 Department of Cardiology, Erasmus MC, Rotterdam, The Netherlands

**Keywords:** Chronic kidney disease, Cardiac remodeling, Proteomics, Coronary flow reserve, Oxidative stress

## Abstract

Chronic kidney disease (CKD) predisposes to cardiac remodeling and coronary microvascular dysfunction. Studies in swine identified changes in microvascular structure and function, as well as changes in mitochondrial structure and oxidative stress. However, CKD was combined with metabolic derangement, thereby obscuring the contribution of CKD alone. Therefore, we studied the impact of CKD on the heart and combined proteome studies with measurement of cardiac function and perfusion to identify processes involved in cardiac remodeling in CKD. CKD was induced in swine at 10–12 weeks of age while sham-operated swine served as controls. 5–6 months later, left ventricular (LV) function and coronary flow reserve were measured. LC–MS–MS-based proteomic analysis of LV tissue was performed. LV myocardium and kidneys were histologically examined for interstitial fibrosis and oxidative stress. Renal embolization resulted in mild chronic kidney injury (increased fibrosis and urinary NGAL). PV loops showed LV dilation and increased wall stress, while preload recruitable stroke work was impaired in CKD. Quantitative proteomic analysis of LV myocardium and STRING pre-ranked functional analysis showed enrichments in pathways related to contractile function, reactive oxygen species, and extracellular matrix (ECM) remodeling, which were confirmed histologically and associated with impaired total anti-oxidant capacity. H_2_O_2_ exposure of myocardial slices from CKD, but not normal swine, impaired contractile function. Furthermore, in CKD, mitochondrial proteins were downregulated suggesting mitochondrial dysfunction which was associated with higher basal coronary blood flow. Thus, mild CKD induces alterations in mitochondrial proteins along with contractile proteins, oxidative stress and ECM remodeling, that were associated with changes in cardiac function and perfusion.

**Supplementary Information:**

The online version contains supplementary material available at 10.1007/s00395-024-01085-7.

## Introduction

Cardiovascular disease is highly prevalent in patients with chronic kidney disease (CKD) and responsible for approximately half of CKD-related deaths [49]. The mechanisms underlying the detrimental impact of CKD on the heart are still incompletely understood, but CKD is associated with endocrine, inflammatory and hemodynamic changes that impact the heart and may contribute to the development of uremic cardiomyopathy [27, 52].

Several animal models have been established to induce primary renal failure to assess the initiation and development of cardiac dysfunction. However, most of these models fail to elucidate the early impact of mild to moderate kidney injury, which goes often undetected in the general population. Moreover, most of the studies are performed in rodents, which do not reflect the full spectrum of the pathophysiology in human heart.

We [45, 53, 54] and others [6, 12, 13] have used swine as a model to assess the impact of CKD on the heart and found reductions in coronary flow reserve (CFR) and perturbations in myocardial perfusion and oxygen delivery [17, 53, 54]. In further support of a central role of coronary microvascular changes, Chade and co-workers found changes in microvascular structure and function as well as changes in expression of genes associated with vascular endothelial growth factor (VEGF) signaling post CKD[12, 13]. Yet, consistent with recent findings in human CKD patients [23, 37], the reduction in CFR was due to an increase in basal coronary blood flow (CBF) rather than a decrease in maximal CBF, suggesting alterations in myocardial metabolism and/or efficiency. In other studies in swine, changes in gene expression associated with fatty acid oxidation were found that were accompanied by changes in mitochondrial structure [6] as well as oxidative stress [13]. More recently, we observed that this oxidative stress was actually beneficial for myocardial perfusion, as alleviation of oxidative stress with the superoxide dismutase mimetic Tempol and the superoxide scavenger MPG resulted in a mild increase in myocardial oxygen extraction during exercise suggestive of coronary microvascular vasoconstriction [11]. However, these porcine studies all combined CKD with metabolic derangement, induced by atherogenic diet and/or diabetes that may directly affect mitochondrial function, thereby confounding the effect of CKD alone.

In the present study, we aimed to focus on the impact of early CKD on cardiac function and perfusion using a swine model of chronic kidney injury, in the absence of any metabolic derangement, to discern the effects of this insult on the heart. We employed our previously established method of renal embolization to cause kidney injury [45, 53] but in the absence of metabolic alterations. We combined hemodynamic analyses of left ventricular function and perfusion with histopathological analysis and unbiased proteomic analysis of left ventricular tissue to assess underlying mechanisms followed by targeted molecular analyses with a focus on markers for endothelial function and oxidative stress.

To further assess a relation between oxidative stress and contractile dysfunction, cultured myocardial slices from swine with and without CKD were exposed to H_2_O_2_ and contractility was measured every 24 h for three days. Since H_2_O_2_ induced a reduction in contractile force in slices from CKD swine consistent with an impaired anti-oxidant defense, further experiments were performed exposing slices form CKD swine to the superoxide dismutase mimetic TEMPOL.

## Methods

All animal experiments were approved by the *Regierung von Oberbayern* (ROB-55.2–2532.Vet_02-19–163) at the Institute for Surgical Research at the Walter-Brendel-Centre of Experimental Medicine, Munich, Germany and were performed in accordance with the guidelines from Directive 2010/63/EU of the European Parliament on the protection of animals used for scientific purposes.

### Induction of CKD

Sixteen German Landrace pigs of either sex were used in the experiment out of which 9 served as control (WT) and 7 of them underwent CKD induction.

At 12 weeks of age, swine underwent micro-embolization of afferent glomerular arterioles in both kidneys. The animals were sedated (ketamine 10% (15 mg/kg) (WDT, Garbsen, Germany), azaperone (2 mg/kg) (Stresnil, Elanco, Bad Homburg, Germany) and atropine sulfate (0.02 mg/kg) (Eifelfango, Neuenahr, Germany) against salivation i.m.), followed by Midazolam 15 mg/kg i.v.) (Ratiopharm-Teva, Ulm, Germany), intubated and artificially ventilated (Primus, Dräger, Lübeck, Germany) with a mixture of O_2_ and N_2_ (1:2). Anesthesia and analgesia were maintained by 1–2% (vol/vol) sevoflurane (Sevorane, AbbVie GmbH, Ludwigshafen, Germany) to ventilation and fentanyl (0.005 mg/kg/h i.v.) (Fentadon, Dechra, Aulendorf, Germany) respectively. Arterial access was obtained via a 9F sheath (Cordis, 504-609X) in the right carotid artery, allowing measurement of blood pressure and heart rate. A Swan-Ganz catheter (131F7, Edwards Lifesciences, Irvine, USA) was advanced sequentially in both renal arteries, and the balloon was inflated and 75 mg of polyethylene microspheres (38–42 μm, Cospheric, Santa Barbara, CA, USA) was infused separately into each kidney. Thereafter, the carotid was ligated and the wound was closed. Carprofen (4 mg/kg) was given against post-operative pain (Rimadyl, Zoetis, Berlin, Germany). Amoxicillin antibiosis was administered (Duphamox LA, Zoetis, Berlin, Germany) perioperatively and animals were subsequently allowed to recover.

### Hemodynamic assessment

At 8 months of age, a terminal experiment was performed. Animals were sedated and pre-anesthetized as previously described. Anesthesia was maintained using propofol (7 mg/kg/h) and fentanyl (0.005 mg/kg/h). An echocardiography was performed (Esaote, MyLabX8vet, Neufahrn, Germany) to assess left ventricular (LV), right ventricular (RV) and left atrial (LA) dimensions, at systole and diastole. For right and left sided heart catherization, an 11F venous sheath (Terumo, RS*C11N10NR, Eschborn, Germany) and a 9F arterial sheath (Cordis, 504-609X, Miami Lakes, USA) were placed in the right external jugular vein and left internal carotid artery respectively. The latter was connected to a pressure transducer to continuously monitor arterial blood pressure and heart rate.

Under fluoroscopic guidance (Ziehm Vision, Nuremberg, Germany), a Swan-Ganz catheter (131F7, Edwards Lifesciences, Irvine, USA) was introduced and progressed into the pulmonary artery via the venous sheath to measure the pressure in pulmonary artery (PA), right ventricle (RV), and right atrium (RA) and to measure the pulmonary capillary wedge pressure (PCWP). The cardiac output was assessed by thermodilution.

Using the arterial access sheath, a pressure–volume loop catheter (FDH-7018B-E245A, Transonic, Ithaca, USA) was placed in the LV and PV loops were recorded using LabChart Pro (ADInstruments, Oxford, United Kingdom). Ventilation was briefly halted to obtain baseline PV loops as well as PV loops during preload reduction with a 14F Fogarty balloon (62080814F, Edwards Lifesciences, Irvine, USA) positioned in the inferior vena cava just below the diaphragm. Approximately 10 cardiac cycles recorded from the start of the occlusion were analyzed to obtain end-systolic and end-diastolic LV volumes (ESV, EDV) and pressures (ESP and EDP), and to calculate the end-diastolic pressure–volume relationship, end-systolic pressure–volume relationship, preload recruitable stroke work (slope of the relation between EDV and stroke work), arterial elastance (Ea, ratio of ESP and stroke volume (SV)), as well as cardiac efficiency (ratio of stroke work and pressure–volume area).

Subsequently, the thorax was opened, and a flow probe (3PSB, Transonic, Ithaca, USA) was placed around the proximal left anterior descending (LAD) coronary artery and connected to a computer using a perivascular flow module (TS420, Transonic, Ithaca, USA) and amplifier (16/35, ADInstruments, Oxford, United Kingdom). Baseline coronary blood flow was measured using LabChart Pro. An 6F angiocatheter (670–082-0E, Cordis, Miami Lakes, USA) was introduced into the LAD to infuse the vasodilator adenosine (50 μg/kg/min i.c., 01890, Merck, Taufkirchen, Germany) until maximum coronary blood flow was achieved. Upon completion of the experiments, ventricular fibrillation was induced using a 9 V battery on the surface of the heart, and the heart was excised. Samples were obtained from RA, RV, LA and LV, snap-frozen in liquid nitrogen and stored at -80 °C until further processing. In addition, a tissue block of the LV was processed to culture myocardial tissue slices.

### Myocardial tissue slice cultivation

To assess the ex vivo contractile function, 300 μm thick living myocardial slices (LMS) were made and cultivated in biomimetic cultivation chambers (BMCCs) using the MyoDish cultivation set-up (InVitroSys, Gräfelfing, Germany). A posterior transmural LV sample (4 cm × 4 cm) was obtained and directly placed into cold (4 °C) cardioplegic buffer according to the LMS protocol developed by Fisher et. al. (2019) [14] and described in detail by Hamers et al. (2022) [16]. According to this protocol, myocardial LV slices were cut, prepared and hung into BMCCs in M199 medium (31,150–022, Thermo Fisher, Waltham, USA) (supplemented with 10% Penicillin–Streptomycin (100x) (P0781, Merck, Taufkirchen, Germany), 10% insulin-transferrin-selenium-X (100x) (51,500,056, Thermo Fisher, Waltham, USA), cort20 and β-mercaptoethanol (A1108.0100, AppliChem GmbH, Darmstadt, Germany). The LMS containing BMCCs were placed onto the specific cultivation rocker (60 rpm) in an incubator (21% O_2_, 5% CO_2_, 80% humidity) and electrically stimulated (30 bpm, 50 mA, 3 ms pulse duration) to contract. The twitch force of the LMS was continuously recorded. The cardiac slices equilibrated for 3 days in cultivation before experiments were started. Fresh cultivation medium was exchanged every other day, by removing the BMCCs from the rocker into a laminar flow hood, where medium was aspirated until approximately 0.8 mL remained. 1.6 mL of fresh medium was added to each dish.

After three days of cultivation, LMS (Con or CKD) were treated with 25 µM hydrogen peroxide, 25 µM Tempol or vehicle in order to study the relative contribution of oxidative stress to cardiac function. The treatment doses were chosen based on previous in vitro studies [8, 26]. Hydrogen peroxide/TEMPOL treatment was administered three times at 24 h intervals. The twitch force of all LMS was assessed at 60 min after each treatment, and normalized to the twitch force 24 h prior to the first treatment. After the third treatment, normalized twitch force over the course of the cultivation was compared between groups.

### Real-time quantitative PCR

Subendocardial left ventricular tissue samples and LMS cultured for 7 days were snap-frozen in liquid N_2_. 30 mg of LV tissue or a single slice of 7 × 7 × 0.3 mm was homogenized and mRNA was extracted using the RNeasy Fibrous Tissue Mini kit, Qiagen, Hilden, Germany). cDNA was synthesized using 1000 ng of mRNA and a cDNA kit (M1661, Thermo Fisher, Waltham, USA). Target genes were normalized against HPRT and GAPDH using the StepOne software (Applied Biosystem CA, USA). Relative gene expression was calculated using the delta–delta Ct method. The primers are listed in Supplementary Table 6.

### Western blots

Subendocardial left ventricular tissue samples were homogenized in RIPA buffer supplemented with protease and phosphatase inhibitor cocktail. 30 µg of protein lysates was loaded in precast protein gels (4–20% gradient gel, Bio-Rad) and transferred to a PVDF membrane (Trans-Blot Turbo Mini 0.2 µm PVDF Transfer Pack, Bio Rad). Membrane was blocked in 5% milk in PBST and then incubated with primary antibody overnight (eNOS and VEGF 1:1000; GAPDH – 1:10,000) and secondary antibody (1:5000) in 5% milk in PBST. The images were captured in iBright CL750 (Thermo Fisher Scientific, Waltham, USA) and quantification of bands by densitometry analysis was conducted in Image J Studio software. The antibodies are listed in Supplementary Table 6.

### Enzyme-linked immunosorbent assays

To study the degree of kidney damage caused by renal embolization, neutrophil gelatinase-associated lipocalin (NGAL) levels were determined in urine samples obtained after sacrifice using ELISA (ab207924, Abcam, Berlin, Germany) per the manufacturer’s instructions. Oxidative stress was determined by measuring the 8-Hydroxy-2′-Deoxyguanosine (8-HDG) secreted in the urine samples using ELISA (ab201734 Abcam, Berlin, Germany) per the manufacturer’s instructions.

### Colorimetric urine analysis

To determine the protein level in urine, urine samples obtained after sacrifice were tested using a colorimetric Coomassie protein assay kit according to the manufacturer’s instructions (23,200, Thermo Fisher, Waltham, USA). To assess total antioxidant capacity, 50 mg of LV sample (endocardium-anterior) was suspended in a lysing tube (845-CS-1140250, Innuscreen GmbH, Berlin, Germany) with 750 µL RIPA buffer (89,901, Thermo Fisher, Waltham, USA) and homogenized using a homogenizer (Speedmill Plus, Analytik Jena GmbH, Jena, Germany). Subsequently, a Trolox total antioxidant capacity (TAC) colorimetric assay (ab65329, Abcam, Berlin, Germany) was performed according to the manufacturer’s instructions.

### Proteomics

Sample Preparation for Proteome Analysis: Frozen left subendocardial ventricular heart tissue samples were placed into precooled tubes and cryopulverized in a CP02 Automated Dry Pulverizer (Covaris, Woburn, MA, USA) with an impact level of 5 according to the manufacturer’s instructions. Tissue lysis was performed in 8 M urea/0.5 M NH_4_HCO_3_ with ultrasonication (18 cycles of 10 s) using a Sonopuls HD3200 (Bandelin, Berlin, Germany). Total protein concentration was quantified using a Pierce 660 nm Protein Assay (Thermo Fisher Scientific, Rockford, IL, USA). Fifty micrograms of protein was digested sequentially, first with Lys-C (FUJIFILM Wako Chemicals Europe GmbH, Neuss, Germany) for 4 h and, subsequently, with modified porcine trypsin (Promega, Madison, WI, USA) for 16 h at 37 °C.

Nano-Liquid Chromatography (LC)–Tandem Mass Spectrometry (MS) Analysis and Bioinformatics:

For LC–MS–MS analysis, an UltiMate 3000 nano-LC system connected online to a Q Exactive HF-X instrument (Thermo Fisher Scientific, Waltham, USA) was used. 1 μg of the digest was injected, transferred to a PepMap 100 C18 trap column (100 µm × 2 cm, 5 µM particles, Thermo Fisher Scientific) and then separated on an analytical column (PepMap RSLC C18, 75 µm × 50 cm, 2 µm particles, Thermo Fisher Scientific) at a flow rate of 250 nL/min with a gradient of 5–20% of solvent B for 80 min, followed by a ramp of 9 min to 40%. 0.1% formic acid in water made up solvent A, and 0.1% formic acid in acetonitrile made up solvent B. MS spectra were acquired with data independent acquisition using 50 12 m/z-wide isolation windows in the range of 400–1000 m/z. Protein identification and peptide quantification were carried out using DIAN-NN (1.8.1) [10] and the NCBI RefSeq Sus scrofa database (v.7–5-2020). All statistical analyses and data visualization were performed using R. Prior to statistical analysis, potential contaminants, only identified by site and reverse hits, were excluded. Proteins with at least two peptides detected in at least three samples of each condition were quantified using the MS-EmpiRe algorithm as previously described [2, 15]. The R script used for quantitative analysis is at https://github.com/bshashikadze/pepquantify. Proteins with a Benjamini–Hochberg-corrected *p *value ≤ 0.05 and fold change ≥ 1.3 were regarded as significantly altered for volcano plot and we used corrected *p *value ≤ 0.05 proteins for protein–protein interaction network. Preranked gene set enrichment analysis using STRING was employed to reveal biological processes related to differentially abundant proteins [48]. Signed (based on fold change) and log-transformed *p* values were used as ranking metrics and the false discovery rate was set to 1%. Raw mass spectrometry data and DIA-NN output data have been deposited to the ProteomeXchange Consortium via the PRIDE [38] partner repository with the dataset identifier PXD050994.

### Histology and immunohistochemistry

LV anterior myocardial tissue and kidney samples were cut and fixated in Rotihistofix (Roth, P087.1) and transferred into 70% alcohol after 48 h. After that, the tissue was embedded in paraffin. 3 µm deparaffinized sections were stained with picrosirius red staining solution (Polysciences, Picrosirius Red Stain Kit#24,901) or Gomori silver stain (Abcam, #ab236473). The picrosirius red (PR) staining was analyzed under polarized light, and the amount of birefringence was quantified in ten randomly chosen nonoverlapping fields (× 200 magnification) using QuPath software. Cross-sectional areas of cardiomyocytes with visible nuclei were measured in Gomori staining. For immunostaining, 3 µm deparaffinized LV sections were boiled in Citrate Buffer, pH 6.0, for antigen retrieval. The sections were incubated with primary antibodies for 8-hydroxy-de-guanosine (1:1000 dilution: Abcam, #ab48508) CD31 (1:100 dilution: Thermo-scientific MA5-32,321) and WGA (1:1000 dilution: Thermo -scientific, W21404) overnight. On the following day, the sections were incubated in species specific secondary antibodies (1:500 dilution; Abcam—anti mouse or ab150113- anti rabbit-ab150080) for two hours and sections were mounted with DAPI for nuclear staining. The stained sections were quantified in ten randomly chosen nonoverlapping fields (× 400 magnification) using ImageJ software.

### Statistical analysis

Statistical analysis was performed using a Student’s t-test or Analysis of Variance for Repeated Measurements as appropriate in Graphpad Prism (v10). All data passed the normality test using the Shapiro–Wilk test and data are shown as mean ± SEM.

## Results

**Fig. 1:**
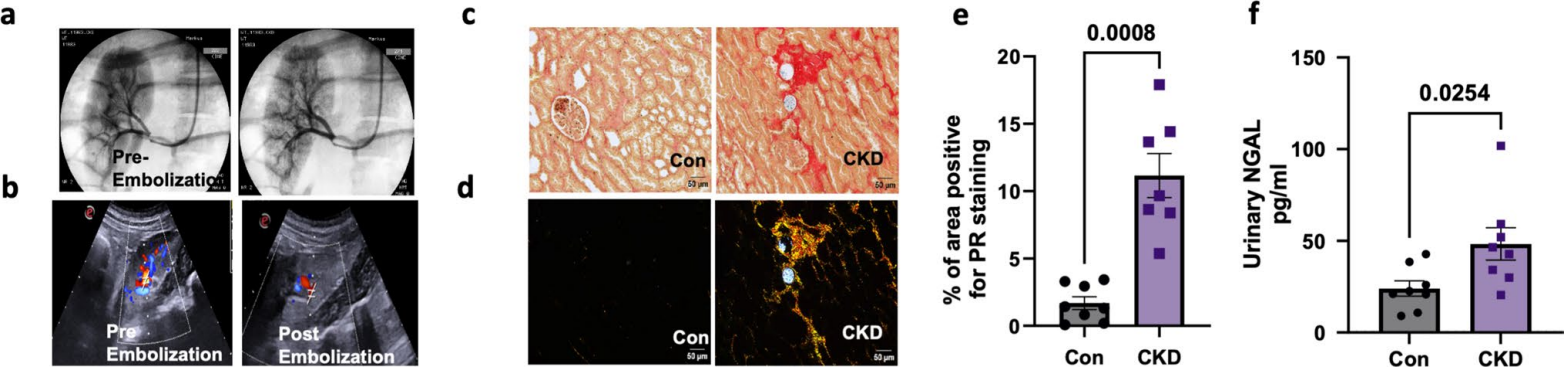
Induction of CKD through renal artery embolization. **a** Angiogram of swine kidney, taken before and after embolization with microspheres into the renal artery, using contrast at 10–12 weeks of age. **b** Representative renal artery flow using ultrasound before and after embolization at 10–12 weeks of age. **c**, **d** Representative images of Picro-sirius red (PR) staining in the kidneys from Con and CKD swine under bright field and polarizing light microscopy respectively post embolization at 8–9 months of age. The blue bubbles in the field indicate microspheres. Original magnification, × 200 **e** Quantification of the percentage of area positive for PR staining under polarizing light microscopy. **f** Quantification of urinary NGAL 5–6 months post embolization. *N* = 8 in Con, *N* = 7 in CKD. Values are mean + / − SEM. *p* value by Student’s *t*-test

Immediately post renal embolization, renal blood flow was reduced as measured by angiography as well as ultrasound (Fig. 1a, b). Post renal embolization at eight months of age, renal blood flow was no longer impaired (Fig S1, a) and the kidney showed significant increase in fibrosis (amount of Picrosirius Red staining) in the areas surrounding the microspheres (Fig. 1c–e) as well as a significant rise in urinary NGAL in the CKD animals (Fig. 1f). However, no proteinuria or changes in urine creatinine levels were observed (Fig S1b, c).

**Fig. 2:**
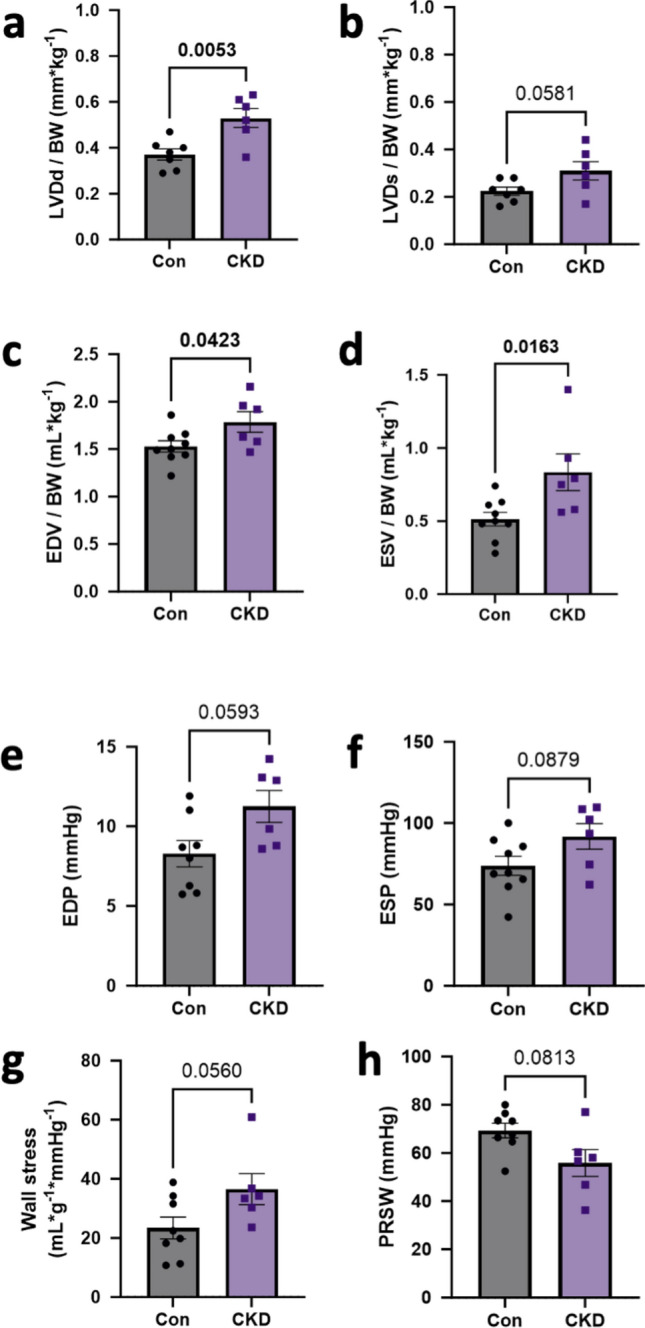
Hemodynamic assessment of the heart post CKD. **a**, **b** LV diastolic diameter (LVDd) and LV systolic diameter (LVDs) respectively, corrected for body weight measured via echocardiography. *N* = 7 animals in Con, *N* = 6 in CKD (**c**,**d**) End-diastolic (EDV) and systolic volume (ESV) corrected for body weight (**e**, **f**) end-diastolic (EDP) and end-systolic pressures (ESP) measured through PV loop. *N* = 9 animals in Con, *N* = 6 in CKD. **g**, **h** Systolic wall stress calculated by end-systolic PV loop measurements and post-mortem LV weight and Preload recruitable stroke work (PRSW). *N* = 8 animals in Con, *N* = 6 in CKD. Values are mean + / − SEM. *p* value by Student’s *t*-test

Overall cardiac function appeared to be preserved, as blood pressure, heart rate (HR), cardiac output (CO) and stroke volume (SV) were unaltered (Supplementary Table S1). Echocardiography revealed increased LV diastolic and systolic diameters (Fig. 2a, b) which was consistent with increased end-diastolic (EDV) and systolic volume (ESV) as measured with PV loop (Fig. 2c, d). Furthermore, PV loop data showed a trend towards an increase in LV end-diastolic pressure (EDP) and systolic pressure (ESP) in CKD swine (Fig. 2e, f). Arterial elastance (Ea) was increased in CKD swine along with significant reduction in the ejection fraction (EF) (Supplementary Table S1). Despite a trend toward left ventricular hypertrophy, this resulted in a trend toward increase in systolic, but not diastolic wall stress in CKD vs. WT-swine (Fig. 2 g; Supplementary Table S1). In addition, contractile function was impaired as evidenced by a significant reduction in the ejection fraction (EF) (Supplementary Table S1) along with significant decrease in preload recruitable stroke work (PRSW), a preload independent measure of contractile function, in the PV loop measurements (Fig. 2 h).

**Fig. 3:**
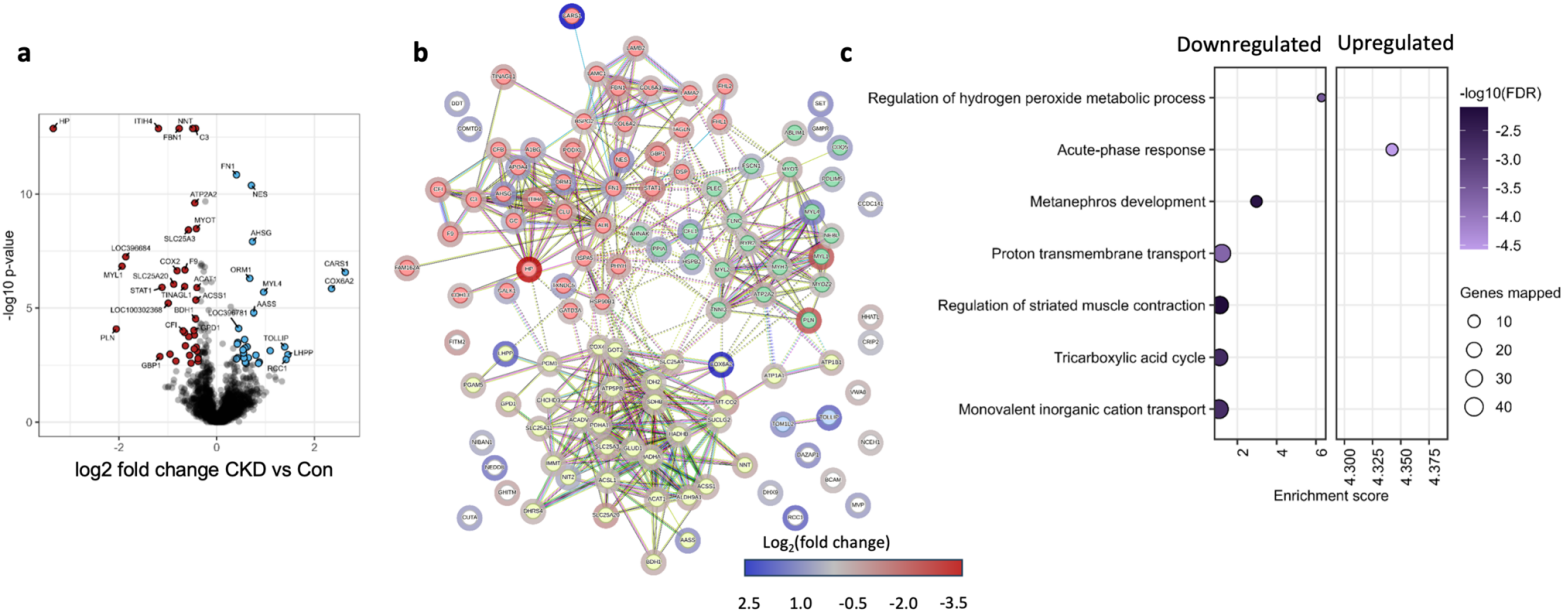
Proteomic analysis of LV endocardial tissue post CKD. **a** Quantitative proteome changes are represented via volcano plots in CKD vs Con animals. Color-filled circles (blue- upregulated, red downregulated, see also color-coding bar) indicate differentially abundant proteins (Benjamini– Hochberg-corrected *p* value < 0.05 and Fold change > 1.3). **b** Pre-ranked proteins according to the fold changes using differentially abundant proteins (*p* value < 0.05) are used to generate a Protein–Protein Interaction network using STRING software. String_kmeans _clustering presented three main clusters. The green-filled circles represent contractile proteins, yellow- mitochondrial, Red- Blood microparticles and ECM proteins. **c** Over-representation analysis using WebGestalt with gene sets according to Kyoto Encyclopedia of Genes and Genomes (KEGG) and gene ontology (GO) biological process databases. Benjamini–Hochberg method was used for multiple testing adjustment. Size of the bubble indicates the corresponding number of differentially abundant proteins (referred to as genes mapped in the figure) and color represents the significance of enrichment. *n* = 5 for Con and *n* = 4 for CKD pigs

In order to investigate the underlying molecular pathways, which are differentially regulated in CKD swine, we performed a label-free liquid chromatography–tandem mass spectrometry analysis (LC–MS/MS) of CKD vs. Control in LV heart samples. Using LC–MS/MS-based proteomics, we identified 3499 proteins with high confidence (false discovery rate < 0.01) (Supplementary Data 1, Table S2). Differential abundance analysis identified 65 proteins significantly different between the two groups (Benjamini–Hochberg-corrected *p *value < 0.05 and fold change ≥ 1.3) which were visualized via volcano plot (Fig. 3a, Table S3). We performed STRING ranked network analysis using fold change for proteins with corrected *p *value < 0.05 and detected 3 main clusters pertaining to contractile proteins, blood microparticles and mitochondrial proteins (Fig. 3b, Table S5). In line with our PV loop data, we observed downregulation of several contractile proteins (MYH7, MYL2, MYL1, MYL4, and TNNI3) as well as differential regulation of proteins involved in Ca^2+^ handling, with downregulation of RYR2 and ATP2A2 (SERCA), and upregulation of Phospholamban (PLN). STRING pre-ranked functional enrichment analysis of proteome profiles from the Gene Ontology (GO) biological processes database revealed 7 downregulated and 1 upregulated significantly enriched terms, respectively with enrichment factor > 1 (Fig. 3c, Table S4), including a downregulation of muscle contraction. Interestingly, the most enriched pathway was for regulation of the hydrogen peroxide (H_2_O_2_) signaling, which was downregulated. In addition, there was also enrichment of the pathways for proton transport and tricarboxylic acid cycle (TCA). These pathways indicated mitochondrial dysfunction in the CKD animals.

**Fig. 4:**
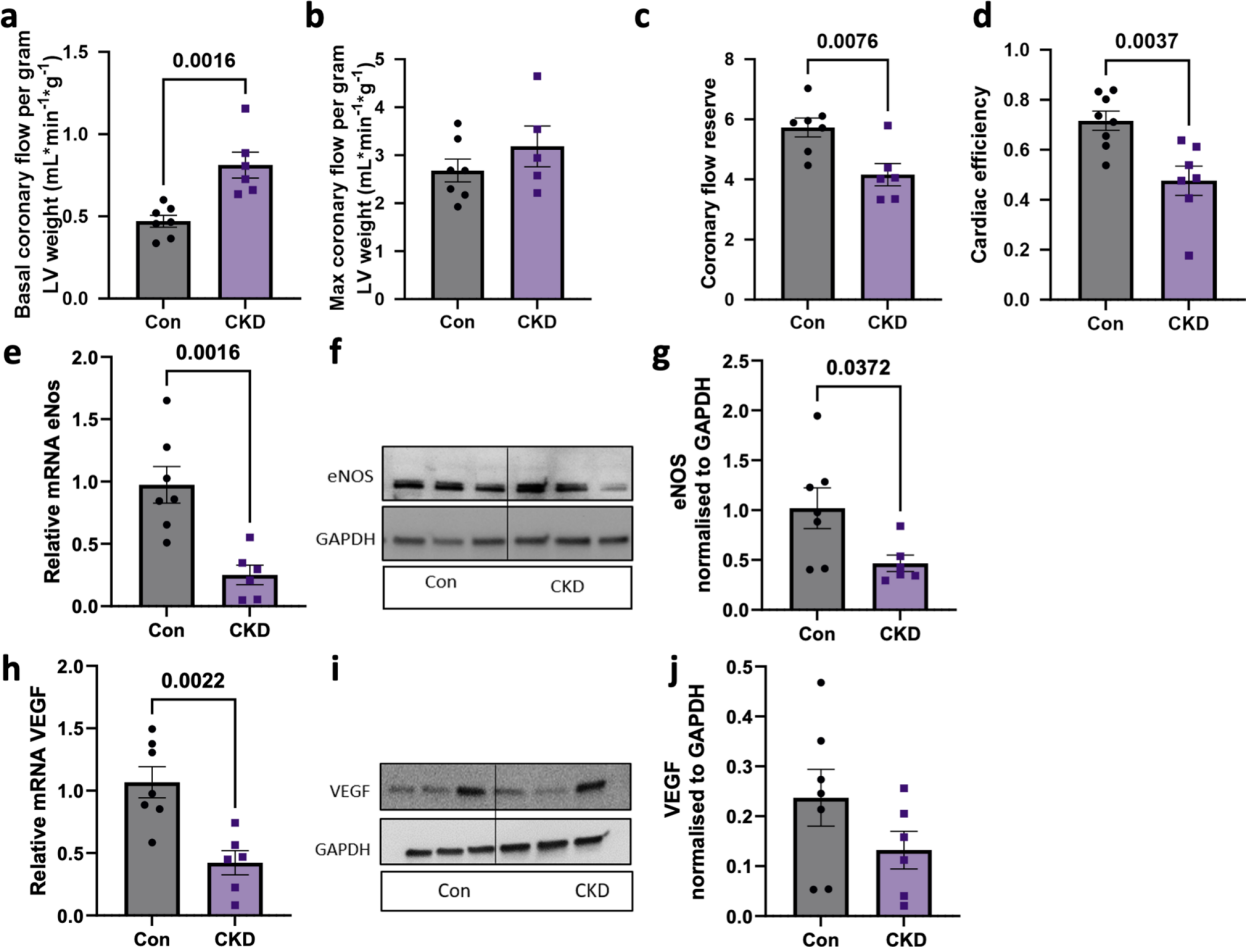
Coronary flow and endothelial function in LV tissue post CKD. **a** Coronary blood flow during baseline, **b** during maximal vasodilation due to adenosine (i.c.) and **c** ratio of maximal flow to baseline flow (Coronary flow reserve) in Con and CKD groups *N* = 8 animals in Con, *N* = 6 in CKD. (d) Cardiac efficiency *N* = 8 animals in WT, *N* = 7 in CKD (**e**–**g**) Quantitative RT-PCR of eNOS, representative blot and quantification of eNOS protein respectively in the LV endocardial tissue in Con and CKD groups. **h**–**j** Quantitative RT-PCR of VEGF, representative blot and quantification of VEGF protein respectively in the LV endocardial tissue in Con and CKD groups. *N* = 7 animals in WT, *N* = 6 in CKD. Values are mean + / − SEM, *p*-value by Student’s *t*-test

Using a flow probe around the left anterior descending coronary artery, we detected an increase in basal coronary blood flow, while maximal coronary blood flow after intracoronary adenosine administration was unaltered, resulting in a decreased coronary flow reserve (Fig. 4a–c). Interestingly, the increase in basal coronary blood flow was associated with a decrease in cardiac efficiency (Fig. 4d) as well as with a decrease in eNOS mRNA as well as protein expression (Fig. 4e–g). We detected similar downregulation in mRNA expression for VEGF but not in protein expression (Fig. 4 h, j).

**Fig. 5:**
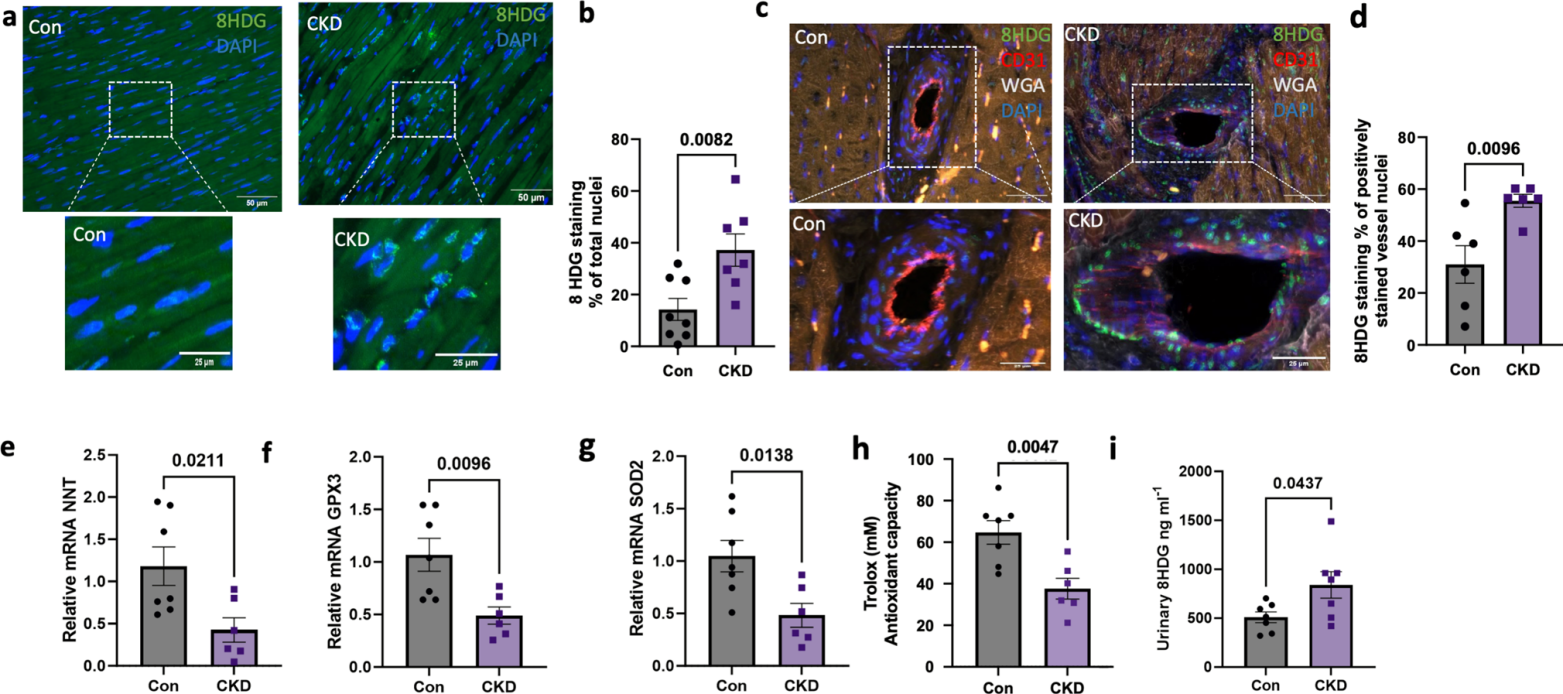
Oxidative stress in LV tissue post CKD. **a** Representative images of immunofluorescence staining for 8-Hydroxy-2'-Deoxyguanosine (8HDG) in whole tissue in LV paraffin-embedded tissue in Con and CKD groups and **b** Quantification of number of nuclei positive for staining of 8HDG. Original magnification, × 400. **c** Representative images of immunofluorescence staining for 8HDG in CD31 stained vessel in LV paraffin-embedded tissue in Con and CKD groups and **d** Quantification of number of nuclei positive for staining of 8-HDG in CD31 stained vessels. Original magnification, × 400 *N* = 8 in Con, *N* = 7 in CKD Scale bar: 50 μm (25 μm for cropped image) **e**–**g** Quantitative RT-PCR of NNT, SOD2, GPX3, in the LV endocardial tissue in Con and CKD groups. **h** Quantification of Trolox for antioxidative capacity in the LV tissue homogenate i) Quantification of secreted 8HDG in the urine at sacrifice. *N* = 7 in Con, *N* = 6 in CKD. Values are mean + / − SEM. *p*-value by Student’s *t*-test

The changes in coronary blood flow and mitochondrial proteins are likely also further connected to increased oxidative stress and associated mitochondrial dysfunction as evidenced by our proteomic data. We confirmed this finding further by an increase in 8-HDG staining in the myocardium as well as the coronary microvasculature (Fig. 5a, d). In the proteomic data, most prominent changes were observed in nicotinamide nucleotide transhydrogenase (NNT) which was downregulated. NNT is an integral component of the mitochondrial inner membrane, producing NADPH, which is subsequently involved in the mitochondrial anti-oxidant defense. Downregulation of NNT was confirmed at the transcriptional levels along with glutathione peroxidase 3 (GPX3) and superoxide dismutase 2 (SOD2, while transcription of other genes involved in anti-oxidant defense, glutathione peroxidase 1 (GPX1) and NADPH oxidase 4 (NOX4) were unchanged (Fig. 5e–g) (Fig S2a, b). Furthermore, we detected lower levels of Trolox as it indicates the antioxidant capacity in the heart post CKD (Fig. 5 h). Finally, for a global measurement of oxidative stress in the animal, we measured the 8-HDG in the urine which was significantly higher post CKD (Fig. 5i).

**Fig. 6:**
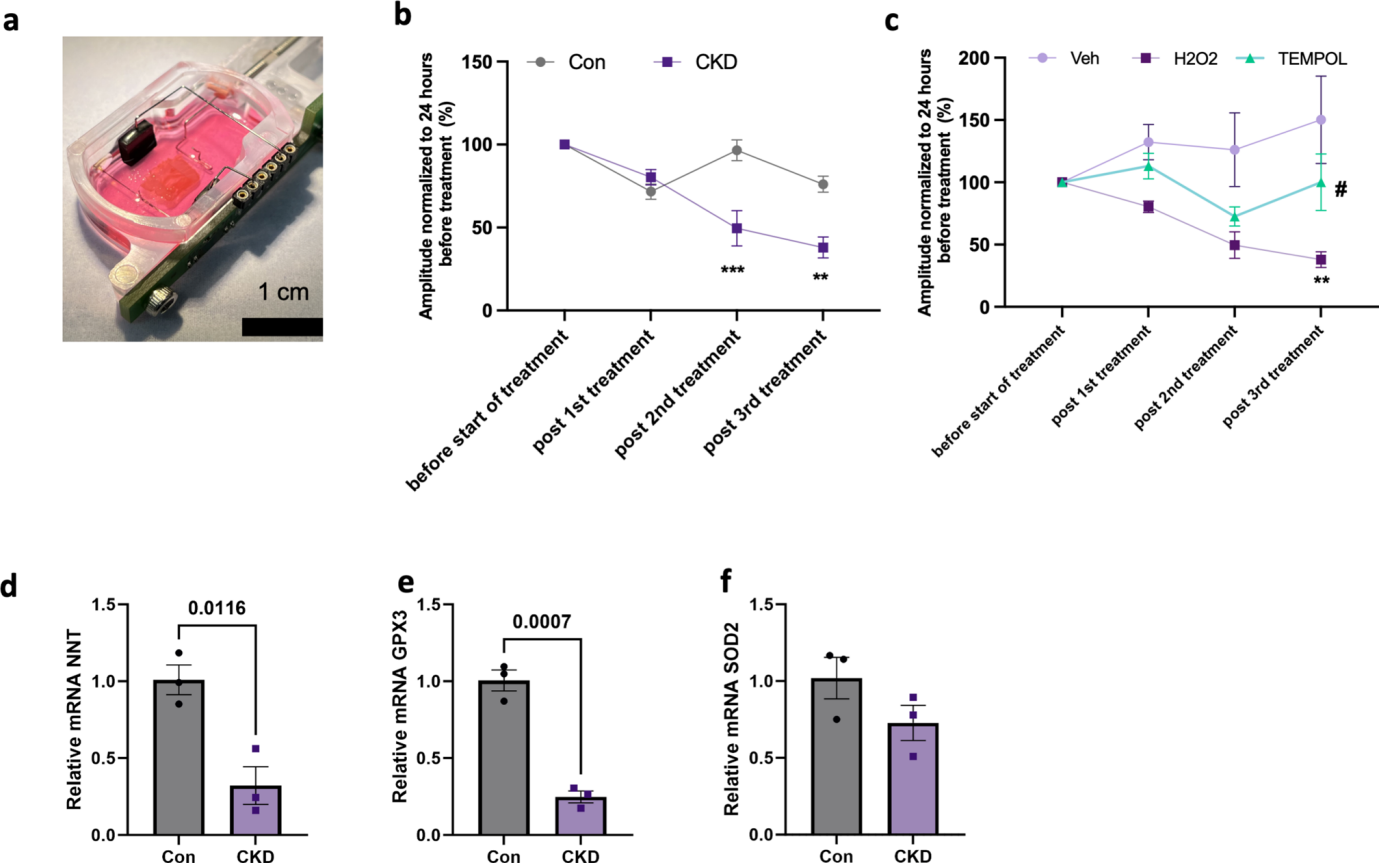
Ex vivo culture of swine Living myocardial slices (LMS). **a** Image of LMS in a biomimetic cultivation chamber (BMCC). **b** LMS from Con and CKD treated with H_2_O_2_ at 24 h intervals. *N* = 3 in Con and *N* = 6 in CKD group ** *p* < 0.01, ***p < 0.001 2- way ANOVA, **c** LMS from CKD treated with Vehicle (Veh), H_2_O_2_, and Tempol at 24 h interval. **p < 0.01 for H_2_O_2_ vs Tempol, ^#^*p* < 0.05 for Tempol vs Veh. 2-way ANOVA. *N* = 3 in Veh; *N* = 4 in TEMPOL and *N* = 6 in H_2_O_2_ group (**c**–**e**) Quantitative RT-PCR of NNT, GPX3 and SOD2 mRNA in LMS from Con and CKD swine. *N* = 3 slices in each group. Values are mean + / − SEM. *p*-value by Student’s *t*-test

Given the fact that regulation of H_2_O_2_ signaling was among the most differentially regulated pathways in CKD, and to further assess a causal relation between CKD, impaired anti-oxidant defense, oxidative stress and changes in cardiac function, living myocardial slices (LMS) obtained from the hearts of swine with CKD as well as control swine were incubated with H_2_O_2_. The contraction amplitude of the LMS was normalized to the contraction amplitude measured 24 h before the start of treatment. Since ROS are short-lived, we compared the contractile amplitude post 60 min of H_2_O_2_ treatment. After 24 and 48 h of incubation with H_2_O_2_, contractile function was depressed in LMS (Fig. 6a, b) from CKD but not control swine. Similar to our findings in myocardial tissue of the swine, this was associated with a lower expression of NNT and GPX3, while the lower levels of SOD2 failed to reach statistical significance (Fig. 6d–f). To delineate a role for endogenous oxidative stress, LMS from CKD swine were incubated with the SOD mimetic Tempol and compared to H_2_O_2_ treatment and vehicle treatment (Veh). Interestingly, TEMPOL caused a reduction in contractile force in the LMS initially, but by the third treatment, the slice contractility started improving significantly compared to the H_2_O_2_ treated LMS (Fig. 6c).

**Fig. 7:**
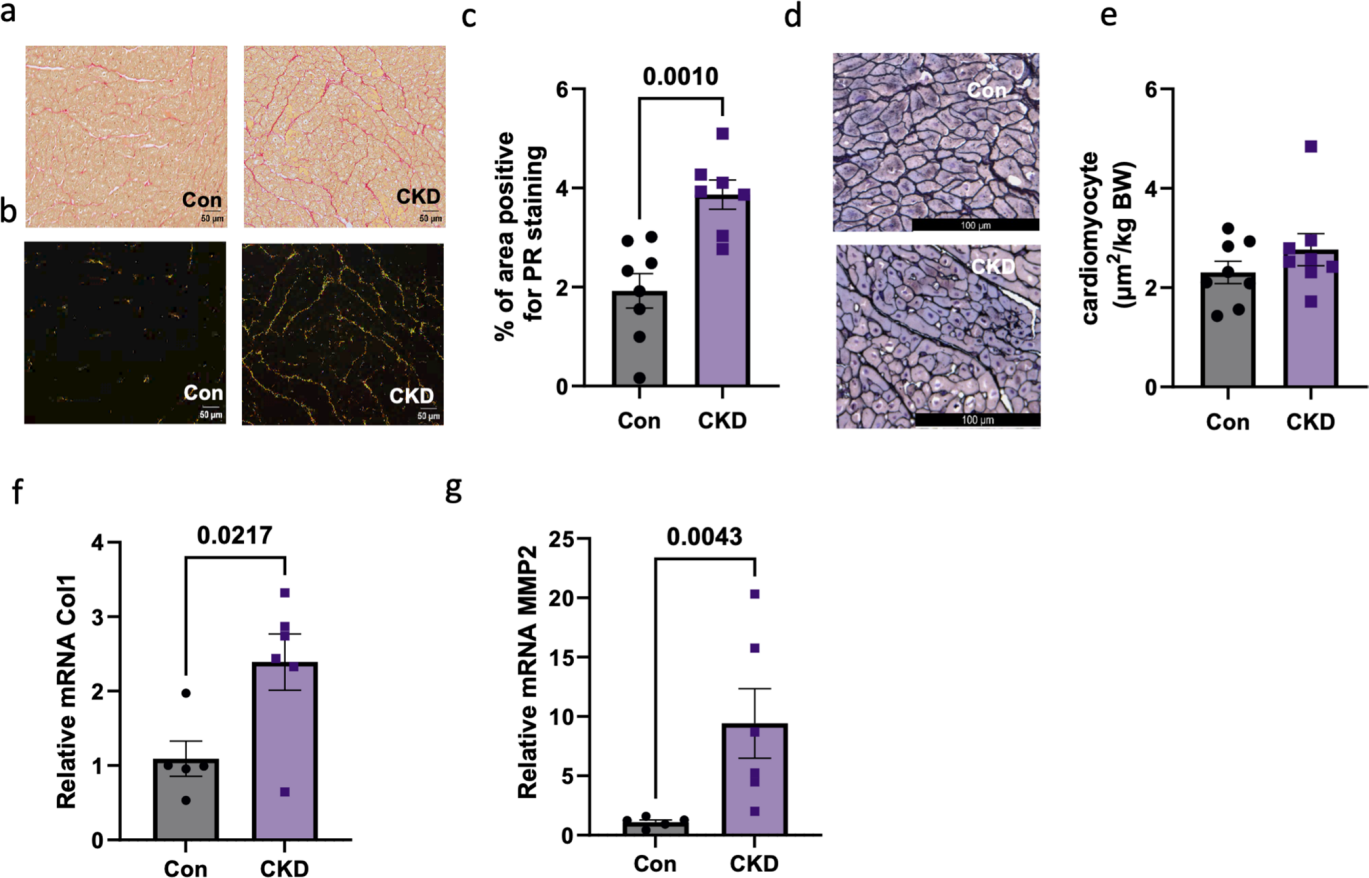
Remodeling in LV tissue post CKD. (a) Representative images of picrosirius red (PR) of the heart under bright field and (b) polarizing light respectively and (c) quantification of the area positive for PR staining under polarizing light (d–e) Quantitative RT-PCR of COl1 and MMP2 mRNA in the LV endocardial tissue in Con and CKD groups. N = 7 animals in Con, N = 6 in CKD. (g) Representative images of Gömöri staining under bright light microscopy for Con and CKD and (h) quantification of the cardiomyocyte area. Original magnification, × 200. Values are mean + / − SEM. p value by Student’s t-test

Finally, PR staining of myocardial tissue showed increased extracellular matrix (ECM) deposition in CKD swine as compared to control (Fig. 7a–f), which was accompanied by changes in collagen 1 (Col1) and metalloprotease 2 (MMP2) gene expression. Furthermore, the increased ECM deposition is in line with proteomics data which also showed upregulation of ECM proteins like Fibrillin 1 (FN1) and the intermediate filament protein Nestin (NES), while FBN1, LAMA2, LAMB2 and LAMC1 were downregulated (red in string plot) in CKD swine (Fig. 2 a,b). Gömöri staining for cardiomyocyte size confirmed the absence of cardiac hypertrophy (Fig. 7g, h).

## Discussion

**Fig. 8:**
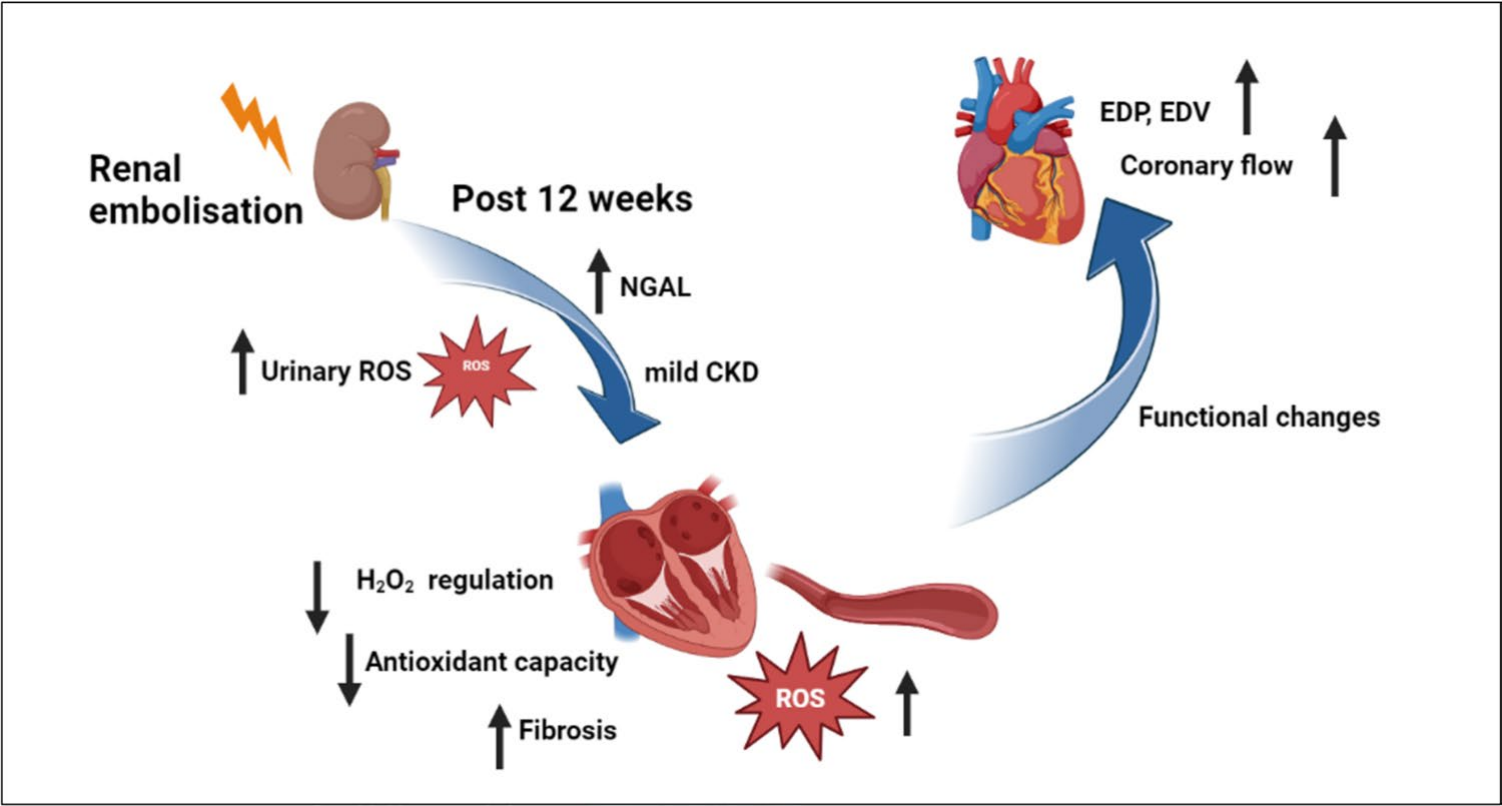
Early molecular and functional changes Post CKD in the heart

Our study's key findings are that early CKD in our swine model increased systemic ROS, which was accompanied by oxidative stress in the myocardial tissue and coronary microvasculature as well as more interstitial fibrosis. This, in turn, led to LV dilatation and contractile dysfunction, which were accompanied by an increase in basal coronary blood flow and a decrease in coronary flow reserve (Fig. 8). In-depth histological and molecular characterization of the LV tissue showed downregulation of contractile proteins, mitochondrial proteins and proteins involved in antioxidant defense that were associated with increased oxidative stress in the myocardium and the coronary microvasculature as well as increased interstitial fibrosis.

Basal ROS levels are necessary for homeostatic functions in the heart, but a pathological increase in ROS level can wreak havoc on normal physiology [18, 35]. Previous work has shown that ROS play an active role in post-kidney damage and contribute to uremic cardiomyopathy [40]. Sub-phenotyping of HFrEF patients revealed that patients with kidney disease and elevated oxidative stress had a worse prognosis than patients with HFrEF who did not have kidney disease and low oxidative stress-signaling [39]. In our model, chronic kidney damage was evidenced by increased urinary NGAL but proteinuria, albuminuria and creatinuria were absent, indicating preserved glomerular function consistent with mild chronic kidney damage. However, we observed significant changes in the cardiac contractile function and coronary flow even with modest damage post embolization. Given that routine screening for cardiovascular function in patients with even mild CKD is recommended in order to prevent CVD, this is noteworthy [36].

The changes that we observed in hemodynamics and the coronary flow in the heart were further investigated via histological and proteomic analyses. Histology showed an increase in 8HDG staining, consistent with oxidative DNA damage in the cardiomyocytes as well as the coronary microvasculature.

In the proteomic analyses, the most prominent changes in CKD pertained to regulation of the H_2_O_2_ signaling pathway and mitochondrial dysfunction, pointing toward a prominent role of the mitochondria as a source for ROS in the heart tissue post CKD. This finding is in agreement with upregulation of the oxidative stress seen in bulk RNA-seq data performed in another swine model of cardiorenal syndrome, albeit with a more severe form of CKD [7]. STRING network as well as pathway analysis indicated downregulation of proteins involved in H_2_O_2_ regulation, proton pump as well as TCA cycle. Interestingly, NNT was the common and most significantly downregulated protein among them (Supplementary data Table S3). NNT, a protein located in the mitochondrial inner membrane, is a key enzyme for the mitochondrial defense system against ROS by producing NADPH [19]. Along with this, we observed significant transcriptional downregulation of several components of mitochondrial redox defense viz. NNT, SOD2, and GPX3 [25].

There are two opposing views when it comes to the role of NNT as an enzyme which regulates the oxidative stress in mitochondria and thereby impacts cellular function. There is ample evidence suggesting the pro-oxidative damage by NNT that is caused in the presence of pathophysiological workload on the heart [32, 34]. Conversely, it has also been shown that loss of NNT makes the heart susceptible to Mn^2+^-dependent superoxide dismutase (Mn-SOD) deletion, leading to cardiomyopathy, as well as to treatment for angiotensin II, which suggests an antioxidative role [21, 24, 28]. Our data in a swine model also argue in favor of an antioxidative role NNT in the heart in presence of mild CKD.

The proteomic data also revealed downregulation of proteins related to contractility post CKD, including sarcomeric proteins (MYL1, MYL2, MYL4, MYH7, and TNNI3), proteins linking the sarcomeres to the Z disks (MYOT, MYOZ2, NEBL, and PDLIM5) as well as proteins involved in calcium handling (RYR2, ATP2A2 (SERCA)), while PLN was upregulated. Downregulation of these proteins post CKD likely contributed to the LV dilation and impaired contractile force observed in vivo. In addition, contractile proteins are susceptible to direct chemical oxidation due to oxidative stress, which can alter their structural conformation and functional activity [4, 46].

To further demonstrate the link between oxidative stress and impaired contractility, we used a novel ex vivo setup for swine living myocardial slice culture with continuous electrical stimulation to preserve the tissue's long-term physiological milieu providing for a perfect setting to examine heart functional changes in response to pro- and anti-oxidants [14, 16]. Indeed, the reduced mRNA levels of both NNT and GPX3, observed in fresh myocardium from CKD swine, persisted in the CKD slices during culture. Upon addition of a low H_2_O_2_ concentration, which reflects a physiological increase in ROS without triggering apoptosis [26], a steady decline in contractility was observed in the CKD slices but not in the Con slices. These data are consistent with other studies that showed the oxidation of thin contractile filament proteins, including actin filament and tropomyosin, in isolated rat hearts, leads to decreased contractility in response to H_2_O_2_ exposure [5]. Contrary to our expectations, adding the SOD mimetic TEMPOL to CKD slices resulted in an initial decline in contractile force. We deduced this is due to production of more endogenous H_2_O_2_ by TEMPOL when encountering superoxide anions in the tissue [3]. With prolonged treatment, the contractility for TEMPOL treated LMS became stronger than those treated with H_2_O_2_ supporting Tempol’s beneficial role in prevention of ROS mediated cardiac remodeling [51].

Mitochondrial dysfunction and oxidative stress in the heart not only influence the cardiac muscle, but the vasculature as well. The present study, using bulk tissue proteomics, cannot distinguish between different cell types, but the majority of protein is derived from cardiomyocytes. As the amount of vascular tissue in the heart is relatively small as compared to cardiac muscle, proteomics is unlikely to be sensitive enough to detect changes. Since nuclei RNA sequencing would be able to delineate changes in different cell types as well as their interactions [29], but is very costly. Therefore, we used RT-PCR and detected a reduction in eNOS and VEGF, which is consistent with recent observations in another CKD swine model [12] and suggestive of endothelial dysfunction. Furthermore, increased oxidative stress in endothelial cells associated with the loss of NNT has been shown to contribute toward eNOS uncoupling [42]. Functionally, coronary flow reserve was impaired albeit mostly due to an increase in basal CBF, rather than a reduction in maximal flow. The reduced flow reserve is also found clinically, in patients with CKD [9, 33, 37, 41] although only one study associated the reduced flow reserve with an increase in basal CBF [33]. The mechanism behind the increase in basal flow is unclear, but may be associated with mitochondrial dysfunction and a switch from NO to H_2_O_2_, as metabolite responsible for vasodilation. Jugulion et al. indeed show such switch from NO to H_2_O_2_ leading to coronary vasodilation in early phase of diabetic cardiomyopathy [22]. Consistent with these data, we found an increase in 8-HDG staining in the coronary microvasculature, indicating increased microvascular oxidative stress. A limitation of the present study is that the impact of oxidative stress on coronary microvascular function was not measured directly. However, we have recently shown in our swine model with multiple comorbidities including CKD that a reduction in oxidative stress through administration of Tempol and MPG resulted in coronary vasoconstriction, suggesting that endogenous H_2_O_2_ acts as a coronary vasodilator in these models [11]. Indeed, previous studies show that H_2_O_2_ released from the endothelium can serve as a mediator for shear stress, acetylcholine- and bradykinin-induced vasodilation in human coronary arterioles[43], rat mesenteric arterioles [55] and in cerebral circulation [50], respectively. Thus, we propose that reduced bioavailability of NO and increased H_2_O_2_ leading to increased basal flow in the coronaries might be an early indicator of endothelial dysfunction post CKD.

Finally, there is an increasing body of research that indicates that changes in redox state affect the immune complement cascade which can trigger systemic inflammation leading to cardiac fibrosis [20]. Our proteomic data showed alterations in expression of proteins from the basement membrane and ECM proteins like Nestin, Fibrillin 1, Fibronectin, Col 6A, etc. [31]. Our mRNA and histological studies in the heart tissues corroborated these findings with significantly increased Col1 and MMP2, two important proteins for ECM remodeling along with increased PR staining [44, 47]. Interestingly, there is recent evidence that the highly conserved cysteine in the propeptide domain of the MMP2 protein is a target for oxidative modifications by superoxide which disrupts a feedback inhibition and provides a nonproteolytic mechanism to activate MMP2 [30]. This redox-activated MMP-2 has been shown to target sarcomeric proteins such as cTnI during ischemia/reperfusion injury, contributing to oxidative stress-dependent defects in cardiac contractility [1]. Altogether, impaired anti-oxidants post CKD can induce ECM remodeling leading to alterations in cardiac structure and function and thereby contributing to the hemodynamic changes observed in our study.

### Future perspectives

Oxidative stress has been the focus for cardiovascular damage post-kidney injury for quite some time, but clinical trials with anti-oxidants have failed so far to improve cardiac function. This is due, at least in part, to lack of systematic studies into the mechanisms underlying myocardial oxidative damage associated with post-chronic kidney injury. Although a limitation of our study is that mitochondrial function was not measured directly, our data argue strongly in favor of mitochondrial alterations and oxidative damage being the primary drivers in causing contractile dysfunction, heart remodeling and changes in coronary flow post CKD. Recent work of Chade et al. in a model of cardiorenal syndrome showed upregulation of miR-374b-3p that can be modulated to alter mitochondrial ROS production [7]. Such direct interventions of the mitochondrial and oxidative stress signaling pathway may provide a more effective therapeutic approach.

## Conclusion

Our study, combining hemodynamic measurements with histological and molecular assessment of the myocardium and coronary microvasculature, underscores the impact of CKD on myocardial mitochondrial pathways, which shifts the equilibrium between the pro- and antioxidant systems resulting in H_2_O_2_ production and oxidative damage leading to impaired cardiac and microvascular function. Pre-clinical research on a number of anti-oxidative medications did not translate into successful clinical trials, despite encouraging preclinical results. Our data suggest that interventions that can enhance and modulate mitochondrial function rather than block the ROS species, may have a beneficial effect on cardiac performance by decreasing the oxidative stress.

## Supplementary Information

Below is the link to the electronic supplementary material.

Supplementary file1 (XLSX 364 KB)

Supplementary file2 (TIFF 14826 KB)

Supplementary file3 (TIFF 14826 KB)

Supplementary file4 (TIFF 14826 KB)

## Data Availability

The raw mass spectrometry data and DIA-NN output data have been deposited to the ProteomeXchange Consortium via the PRIDE partner repository and Code that is used in the analysis is accessible under https://github.com/bshashikadze/pepquantify. The data that support the findings of this study are available from the corresponding author upon reasonable request.

## References

[1] Ali MAM, Cho WJ, Hudson B, Kassiri Z, Granzier H, Schulz R (2010) Titin is a target of matrix metalloproteinase-2: implications in myocardial ischemia/reperfusion injury. Circulation 122:2039–2047. 10.1161/CIRCULATIONAHA.109.930222 21041693 10.1161/CIRCULATIONAHA.109.930222 PMC3057897

[2] Ammar C, Gruber M, Csaba G, Zimmer R (2019) MS-EmpiRe utilizes peptide-level noise distributions for ultra-sensitive detection of differentially expressed proteins. Mol Cell Proteom: MCP 18:1880–1892. 10.1074/mcp.RA119.001509 31235637 10.1074/mcp.RA119.001509 PMC6731086

[3] Avner BS, Hinken AC, Yuan C, Solaro RJ (2010) H2O2 alters rat cardiac sarcomere function and protein phosphorylation through redox signaling. Am J Physiol - Heart Circ Physiol 299:723–730. 10.1152/ajpheart.00050.2010 10.1152/ajpheart.00050.2010 PMC2944474 20562337

[4] Breitkreuz M, Hamdani N (2015) A change of heart: oxidative stress in governing muscle function? Biophys Rev 7:321–341. 10.1007/s12551-015-0175-5 28510229 10.1007/s12551-015-0175-5 PMC5418422

[5] Canton M, Neverova I, Menabò R, Van Eyk J, Di Lisa F (2004) Evidence of myofibrillar protein oxidation induced by postischemic reperfusion in isolated rat hearts. Am J Physiol Heart Circ Physiol 286:H870–H877. 10.1152/ajpheart.00714.2003 14766672 10.1152/ajpheart.00714.2003

[6] Chade AR, Eirin A (2022) Cardiac micro-RNA and transcriptomic profile of a novel swine model of chronic kidney disease and left ventricular diastolic dysfunction. Am J Physiol Heart Circ Physiol 323:H659–H669. 10.1152/ajpheart.00333.2022 36018756 10.1152/ajpheart.00333.2022 PMC9512116

[7] Chade AR, Sitz R, Kelty TJ, McCarthy E, Tharp DL, Rector RS, Eirin A (2024) Chronic kidney disease and left ventricular diastolic dysfunction (CKD-LVDD) alter cardiac expression of mitochondria-related genes in swine. Transl Res. 10.1016/j.trsl.2023.12.004 38262578 10.1016/j.trsl.2023.12.004 PMC11001533

[8] Chami B, Jeong G, Varda A, Maw AM, Kim HB, Fong GM, Simone M, Rayner BS, Wang XS, Dennis JM, Witting PK (2017) The nitroxide 4-methoxy TEMPO inhibits neutrophil-stimulated kinase activation in H9c2 cardiomyocytes. Arch Biochem Biophys 629:19–35. 10.1016/j.abb.2017.07.001 28688768 10.1016/j.abb.2017.07.001

[9] Charytan DM, Skali H, Shah NR, Veeranna V, Cheezum MK, Taqueti VR, Kato T, Bibbo CR, Hainer J, Dorbala S, Blankstein R, Di Carli MF (2018) Coronary flow reserve is predictive of the risk of cardiovascular death regardless of chronic kidney disease stage. Kidney Int 93:501–509. 10.1016/j.kint.2017.07.025 29032954 10.1016/j.kint.2017.07.025 PMC6381827

[10] Demichev V, Messner CB, Vernardis SI, Lilley KS, Ralser M (2020) DIA-NN: neural networks and interference correction enable deep proteome coverage in high throughput. Nat Methods 17:41–44. 10.1038/s41592-019-0638-x 31768060 10.1038/s41592-019-0638-x PMC6949130

[11] van Drie RWA, van de Wouw J, Zandbergen LM, Dehairs J, Swinnen JV, Mulder MT, Verhaar MC, MaassenVanDenBrink A, Duncker DJ, Sorop O, Merkus D (2024) Vasodilator reactive oxygen species ameliorate perturbed myocardial oxygen delivery in exercising swine with multiple comorbidities. Basic Res Cardiol. 10.1007/s00395-024-01055-z 38796544 10.1007/s00395-024-01055-z PMC11461570

[12] Eirin A, Chade AR (2023) Cardiac epigenetic changes in VEGF signaling genes associate with myocardial microvascular rarefaction in experimental chronic kidney disease. Am J Physiol Heart Circ Physiol 324:H14–H25. 10.1152/ajpheart.00522.2022 36367693 10.1152/ajpheart.00522.2022 PMC9762979

[13] Farahani RA, Yu S, Ferguson CM, Zhu X-Y, Tang H, Jordan KL, Saadiq IM, Herrmann SM, Chade AR, Lerman A, Lerman LO, Eirin A (2022) Renal revascularization attenuates myocardial mitochondrial damage and improves diastolic function in pigs with metabolic syndrome and renovascular hypertension. J Cardiovasc Transl Res 15:15–26. 10.1007/s12265-021-10155-3 34269985 10.1007/s12265-021-10155-3 PMC8761225

[14] Fischer C, Milting H, Fein E, Reiser E, Lu K, Seidel T, Schinner C, Schwarzmayr T, Schramm R, Tomasi R, Husse B, Cao-Ehlker X, Pohl U, Dendorfer A (2019) Long-term functional and structural preservation of precision-cut human myocardium under continuous electromechanical stimulation in vitro. Nat Commun 10:1–12. 10.1038/s41467-018-08003-1 30631059 10.1038/s41467-018-08003-1 PMC6328583

[15] Flenkenthaler F, Ländström E, Shashikadze B, Backman M, Blutke A, Philippou-Massier J, Renner S, Hrabe de Angelis M, Wanke R, Blum H, Arnold GJ, Wolf E, Fröhlich T (2021) Differential effects of insulin-deficient diabetes mellitus on visceral vs. subcutaneous adipose tissue-multi-omics insights from the Munich MIDY pig model. Front Med 8:751277. 10.3389/fmed.2021.751277 10.3389/fmed.2021.751277 PMC8650062 34888323

[16] Hamers J, Sen P, Merkus D, Seidel T, Lu K, Dendorfer A (2022) Preparation of human myocardial tissue for long-term cultivation. JoVE J Vis Exp 184. 10.3791/63964 10.3791/63964 35723462

[17] Heusch G (2022) Coronary blood flow in heart failure: cause, consequence and bystander. Basic Res Cardiol 117:1. 10.1007/s00395-022-00909-8 35024969 10.1007/s00395-022-00909-8 PMC8758654

[18] Heusch G, Andreadou I, Bell R, Bertero E, Botker H-E, Davidson SM, Downey J, Eaton P, Ferdinandy P, Gersh BJ, Giacca M, Hausenloy DJ, Ibanez B, Krieg T, Maack C, Schulz R, Sellke F, Shah AM, Thiele H, Yellon DM, Di Lisa F (2023) Health position paper and redox perspectives on reactive oxygen species as signals and targets of cardioprotection. Redox Biol 67:102894. 10.1016/j.redox.2023.102894 37839355 10.1016/j.redox.2023.102894 PMC10590874

[19] Ho H-Y, Lin Y-T, Lin G, Wu P-R, Cheng M-L (2017) Nicotinamide nucleotide transhydrogenase (NNT) deficiency dysregulates mitochondrial retrograde signaling and impedes proliferation. Redox Biol 12:916–928. 10.1016/j.redox.2017.04.035 28478381 10.1016/j.redox.2017.04.035 PMC5426036

[20] Hof A, Geißen S, Singgih K, Mollenhauer M, Winkels H, Benzing T, Baldus S, Hoyer FF (2022) Myeloid leukocytes’ diverse effects on cardiovascular and systemic inflammation in chronic kidney disease. Basic Res Cardiol 117:38. 10.1007/s00395-022-00945-4 35896846 10.1007/s00395-022-00945-4 PMC9329413

[21] Huang T-T, Naeemuddin M, Elchuri S, Yamaguchi M, Kozy HM, Carlson EJ, Epstein CJ (2006) Genetic modifiers of the phenotype of mice deficient in mitochondrial superoxide dismutase. Hum Mol Genet 15:1187–1194. 10.1093/hmg/ddl034 16497723 10.1093/hmg/ddl034

[22] Juguilon C, Wang Z, Wang Y, Enrick M, Jamaiyar A, Xu Y, Gadd J, Chen C-LW, Pu A, Kolz C, Ohanyan V, Chen Y-R, Hardwick J, Zhang Y, Chilian WM, Yin L (2022) Mechanism of the switch from NO to H(2)O(2) in endothelium-dependent vasodilation in diabetes. Basic Res Cardiol 117:2. 10.1007/s00395-022-00910-1 35024970 10.1007/s00395-022-00910-1 PMC8886611

[23] Kashioulis P, Guron CW, Svensson MK, Hammarsten O, Saeed A, Guron G (2020) Patients with moderate chronic kidney disease without heart disease have reduced coronary flow velocity reserve. ESC Heart Fail 7:2797–2806. 10.1002/ehf2.12878 32648666 10.1002/ehf2.12878 PMC7524098

[24] Kim A, Chen C-H, Ursell P, Huang T-T (2010) Genetic modifier of mitochondrial superoxide dismutase-deficient mice delays heart failure and prolongs survival. Mamm Genome 21:534–542. 10.1007/s00335-010-9299-x 21069343 10.1007/s00335-010-9299-x

[25] Krengel U, Törnroth-Horsefield S (2015) Biochemistry. Coping with oxidative stress. Science (New York, NY) 347:125–126. 10.1126/science.aaa3602 10.1126/science.aaa3602 25574006

[26] Kwon SH, Pimentel DR, Remondino A, Sawyer DB, Colucci WS (2003) H(2)O(2) regulates cardiac myocyte phenotype via concentration-dependent activation of distinct kinase pathways. J Mol Cell Cardiol 35:615–621. 10.1016/s0022-2828(03)00084-1 12788379 10.1016/s0022-2828(03)00084-1

[27] Lekawanvijit S (2018) Cardiotoxicity of uremic toxins: a driver of cardiorenal syndrome. Toxins 10:352. 10.3390/toxins10090352 30200452 10.3390/toxins10090352 PMC6162485

[28] Leskov I, Neville A, Shen X, Pardue S, Kevil CG, Granger DN, Krzywanski DM (2017) Nicotinamide nucleotide transhydrogenase activity impacts mitochondrial redox balance and the development of hypertension in mice. J Am Soc Hypertens : JASH 11:110–121. 10.1016/j.jash.2016.12.002 28087333 10.1016/j.jash.2016.12.002

[29] Lother A, Kohl P (2023) The heterocellular heart: identities, interactions, and implications for cardiology. Basic Res Cardiol 118. 10.1007/s00395-023-01000-6 10.1007/s00395-023-01000-6 PMC10371928 37495826

[30] Lovett DH, Mahimkar R, Raffai RL, Cape L, Maklashina E, Cecchini G, Karliner JS (2012) A novel intracellular isoform of matrix metalloproteinase-2 induced by oxidative stress activates innate immunity. PLoS ONE 7:e34177. 10.1371/journal.pone.0034177 22509276 10.1371/journal.pone.0034177 PMC3317925

[31] Martins SG, Zilhão R, Thorsteinsdóttir S, Carlos AR (2021) Linking oxidative stress and DNA damage to changes in the expression of extracellular matrix components. Front Genet 12:673002. 10.3389/fgene.2021.673002 34394183 10.3389/fgene.2021.673002 PMC8358603

[32] Müller M, Bischof C, Kapries T, Wollnitza S, Liechty C, Geißen S, Schubert T, Opacic D, Gerçek M, Fortmeier V, Dumitrescu D, Schlomann U, Sydykov A, Petrovic A, Gnatzy-Feik L, Milting H, Schermuly RT, Friedrichs K, Rudolph V, Klinke A (2022) Right heart failure in mice upon pressure overload is promoted by mitochondrial oxidative stress. JACC Basic Transl Sci 7:658–677. 10.1016/j.jacbts.2022.02.018 35958691 10.1016/j.jacbts.2022.02.018 PMC9357563

[33] Nelson AJ, Puri R, Nicholls SJ, Dundon BK, Richardson JD, Sidharta SL, Teo KS, Worthley SG, Worthley MI (2019) Aortic distensibility is associated with both resting and hyperemic coronary blood flow. Am J Physiol Heart Circ Physiol 317:H811–H819. 10.1152/ajpheart.00067.2019 31441693 10.1152/ajpheart.00067.2019

[34] Nickel AG, von Hardenberg A, Hohl M, Löffler JR, Kohlhaas M, Becker J, Reil J-C, Kazakov A, Bonnekoh J, Stadelmaier M, Puhl S-L, Wagner M, Bogeski I, Cortassa S, Kappl R, Pasieka B, Lafontaine M, Lancaster CRD, Blacker TS, Hall AR, Duchen MR, Kästner L, Lipp P, Zeller T, Müller C, Knopp A, Laufs U, Böhm M, Hoth M, Maack C (2015) Reversal of mitochondrial transhydrogenase causes oxidative stress in heart failure. Cell Metab 22:472–484. 10.1016/j.cmet.2015.07.008 26256392 10.1016/j.cmet.2015.07.008

[35] Nishida M, Maruyama Y, Tanaka R, Kontani K, Nagao T, Kurose H (2000) G alpha(i) and G alpha(o) are target proteins of reactive oxygen species. Nature 408:492–495. 10.1038/35044120 11100733 10.1038/35044120

[36] Ortiz A, Wanner C, Gansevoort R (2022) Chronic kidney disease as cardiovascular risk factor in routine clinical practice: a position statement by the Council of the European Renal Association. Eur J Prev Cardiol 29:2211–2215. 10.1093/eurjpc/zwac186 35997796 10.1093/eurjpc/zwac186

[37] Park S, Lee SH, Shin D, Hong D, Joh HS, Choi KH, Kim HK, Ha SJ, Park TK, Yang JH, Bin SY, Hahn J-Y, Choi S-H, Gwon H-C, Lee JM (2023) Prognostic impact of coronary flow reserve in patients with CKD. Kidney Int Rep 8:64–74. 10.1016/j.ekir.2022.10.003 36644355 10.1016/j.ekir.2022.10.003 PMC9832048

[38] Perez-Riverol Y, Bai J, Bandla C, García-Seisdedos D, Hewapathirana S, Kamatchinathan S, Kundu DJ, Prakash A, Frericks-Zipper A, Eisenacher M, Walzer M, Wang S, Brazma A, Vizcaíno JA (2022) The PRIDE database resources in 2022: a hub for mass spectrometry-based proteomics evidences. Nucleic Acids Res 50:D543–D552. 10.1093/nar/gkab1038 34723319 10.1093/nar/gkab1038 PMC8728295

[39] Petersen TB, de Bakker M, Asselbergs FW, Harakalova M, Akkerhuis KM, Brugts JJ, van Ramshorst J, Lumbers RT, Ostroff RM, Katsikis PD, van der Spek PJ, Umans VA, Boersma E, Rizopoulos D, Kardys I (2023) HFrEF subphenotypes based on 4210 repeatedly measured circulating proteins are driven by different biological mechanisms. EBioMedicine 93:104655. 10.1016/j.ebiom.2023.104655 37327673 10.1016/j.ebiom.2023.104655 PMC10279550

[40] Podkowińska A, Formanowicz D (2020) Chronic kidney disease as oxidative stress-and inflammatory-mediated cardiovascular disease. Antioxidants 9:1–54. 10.3390/antiox9080752 10.3390/antiox9080752 PMC7463588 32823917

[41] Radhakrishnan A, Pickup LC, Price AM, Law JP, McGee KC, Fabritz L, Senior R, Steeds RP, Ferro CJ, Townend JN (2021) Coronary microvascular dysfunction is associated with degree of anaemia in end-stage renal disease. BMC Cardiovasc Disord 21:211. 10.1186/s12872-021-02025-2 33902440 10.1186/s12872-021-02025-2 PMC8074270

[42] Rao KNS, Shen X, Pardue S, Krzywanski DM (2020) Nicotinamide nucleotide transhydrogenase (NNT) regulates mitochondrial ROS and endothelial dysfunction in response to angiotensin II. Redox Biol 36:101650. 10.1016/j.redox.2020.101650 32763515 10.1016/j.redox.2020.101650 PMC7408723

[43] SenthilKumar G, Katunaric B, Zirgibel Z, Lindemer B, Jaramillo-Torres MJ, Bordas-Murphy H, Schulz ME, Pearson PJ, Freed JK (2024) Necessary role of ceramides in the human microvascular endothelium during health and disease. Circ Res 134:81–96. 10.1161/CIRCRESAHA.123.323445 38037825 10.1161/CIRCRESAHA.123.323445 PMC10766100

[44] Sevin G, Ozsarlak-Sozer G, Keles D, Gokce G, Reel B, Ozgur HH, Oktay G, Kerry Z (2013) Taurine inhibits increased MMP-2 expression in a model of oxidative stress induced by glutathione depletion in rabbit heart. Eur J Pharmacol 706:98–106. 10.1016/j.ejphar.2013.02.052 23500209 10.1016/j.ejphar.2013.02.052

[45] Sorop O, Heinonen I, van Kranenburg M, van de Wouw J, de Beer VJ, Nguyen ITN, Octavia Y, van Duin RWB, Stam K, van Geuns R-J, Wielopolski PA, Krestin GP, van den Meiracker AH, Verjans R, van Bilsen M, Danser AHJ, Paulus WJ, Cheng C, Linke WA, Joles JA, Verhaar MC, van der Velden J, Merkus D, Duncker DJ (2018) Multiple common comorbidities produce left ventricular diastolic dysfunction associated with coronary microvascular dysfunction, oxidative stress, and myocardial stiffening. Cardiovasc Res 114:954–964. 10.1093/cvr/cvy038 29432575 10.1093/cvr/cvy038 PMC5967461

[46] Steinberg SF (2013) Oxidative stress and sarcomeric proteins. Circ Res 112:393–405. 10.1161/CIRCRESAHA.111.300496 23329794 10.1161/CIRCRESAHA.111.300496 PMC3595003

[47] Sygitowicz G, Maciejak-Jastrzębska A, Sitkiewicz D (2021) A review of the molecular mechanisms underlying cardiac fibrosis and atrial fibrillation. J Clin Med. 10.3390/jcm10194430 34640448 10.3390/jcm10194430 PMC8509789

[48] Szklarczyk D, Gable AL, Lyon D, Junge A, Wyder S, Huerta-Cepas J, Simonovic M, Doncheva NT, Morris JH, Bork P, Jensen LJ, von Mering C (2019) STRING v11: protein-protein association networks with increased coverage, supporting functional discovery in genome-wide experimental datasets. Nucleic Acids Res 47:D607–D613. 10.1093/nar/gky1131 30476243 10.1093/nar/gky1131 PMC6323986

[49] Uduman J (2018) Epidemiology of cardiorenal syndrome. Adv Chronic Kidney Dis 25:391–399. 10.1053/j.ackd.2018.08.009 30309456 10.1053/j.ackd.2018.08.009

[50] Wei EP, Kontos HA (1990) H2O2 and endothelium-dependent cerebral arteriolar dilation. Implications for the identity of endothelium-derived relaxing factor generated by acetylcholine. Hypertension 16:162–169. 10.1161/01.hyp.16.2.162 2379949 10.1161/01.hyp.16.2.162

[51] Wilcox CS (2010) Effects of tempol and redox-cycling nitroxides in models of oxidative stress. Pharmacol Ther 126:119–145. 10.1016/j.pharmthera.2010.01.003 20153367 10.1016/j.pharmthera.2010.01.003 PMC2854323

[52] van de Wouw J, Broekhuizen M, Sorop O, Joles JA, Verhaar MC, Duncker DJ, Danser AHJ, Merkus D (2019) Chronic kidney disease as a risk factor for heart failure with preserved ejection fraction: a focus on microcirculatory factors and therapeutic targets. Front Physiol. 10.3389/fphys.2019.01108 31551803 10.3389/fphys.2019.01108 PMC6737277

[53] van de Wouw J, Sorop O, van Drie RWA, van Duin RWB, Nguyen ITN, Joles JA, Verhaar MC, Merkus D, Duncker DJ (2020) Perturbations in myocardial perfusion and oxygen balance in swine with multiple risk factors: a novel model of ischemia and no obstructive coronary artery disease. Basic Res Cardiol 115:1–18. 10.1007/s00395-020-0778-2 10.1007/s00395-020-0778-2 PMC7042191 32100119

[54] van de Wouw J, Sorop O, van Drie RWA, Joles JA, Danser AHJ, Verhaar MC, Merkus D, Duncker DJ (2021) Reduced nitric oxide bioavailability impairs myocardial oxygen balance during exercise in swine with multiple risk factors. Basic Res Cardiol 116:50. 10.1007/s00395-021-00890-8 34435256 10.1007/s00395-021-00890-8 PMC8387273

[55] Zhou X, Bohlen HG, Miller SJ, Unthank JL (2008) NAD(P)H oxidase-derived peroxide mediates elevated basal and impaired flow-induced NO production in SHR mesenteric arteries in vivo. Am J Physiol Heart Circ Physiol 295:H1008–H1016. 10.1152/ajpheart.00114.2008 18599598 10.1152/ajpheart.00114.2008 PMC2544510

