# Supplementary figures and images for: Oxidative stress initiates hemodynamic change in CKD-induced heart disease
Source: Basic Res Cardiol. 2024 Oct 15;119(6):957–71. doi: 10.1007/s00395-024-01085-7 (PMC11628585; doi:10.1007/s00395-024-01085-7)

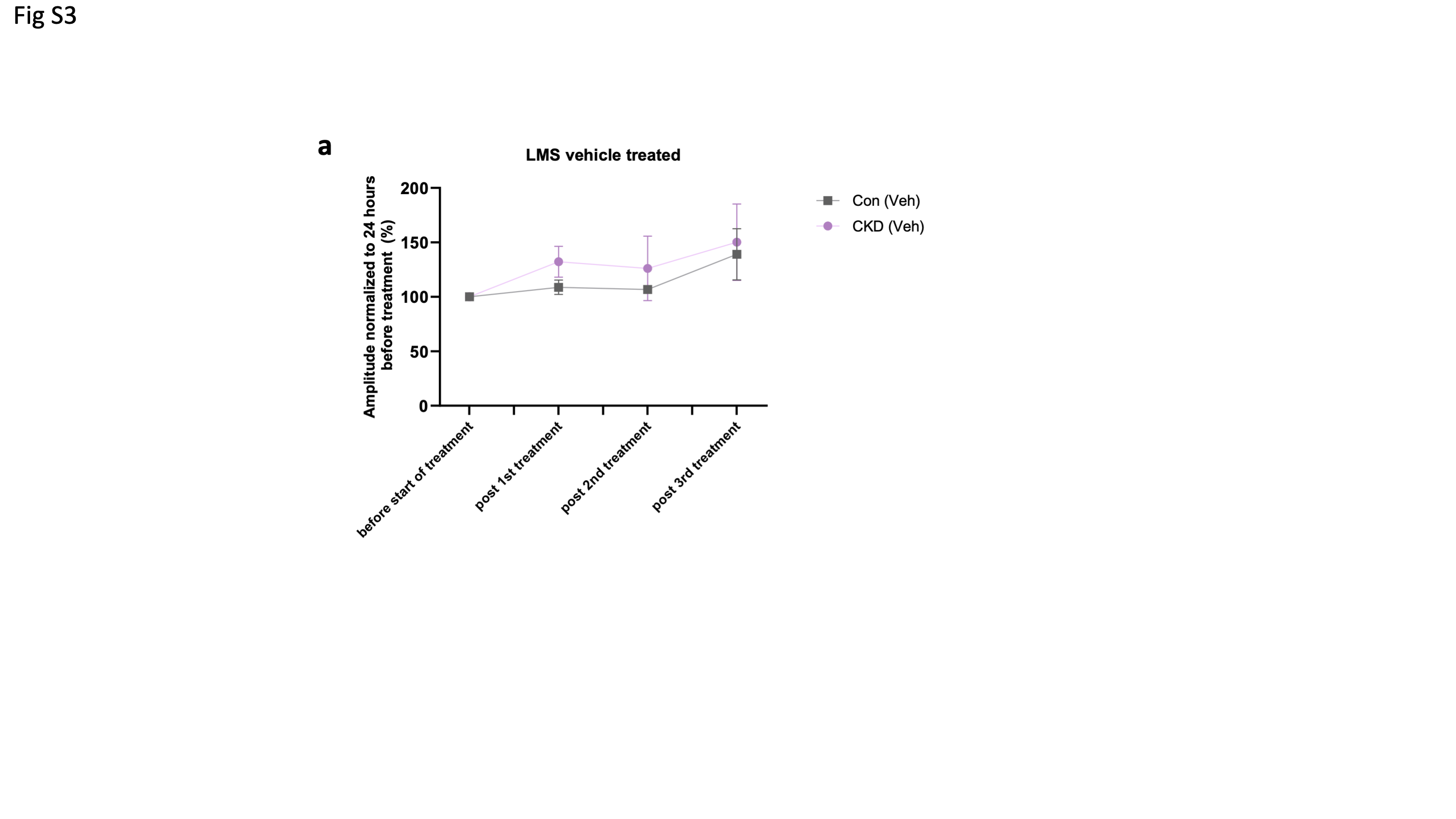

Supplement: Supplementary file 2 — Supplementary file2 (TIFF 14826 KB) [file 395_2024_1085_MOESM2_ESM.tiff]

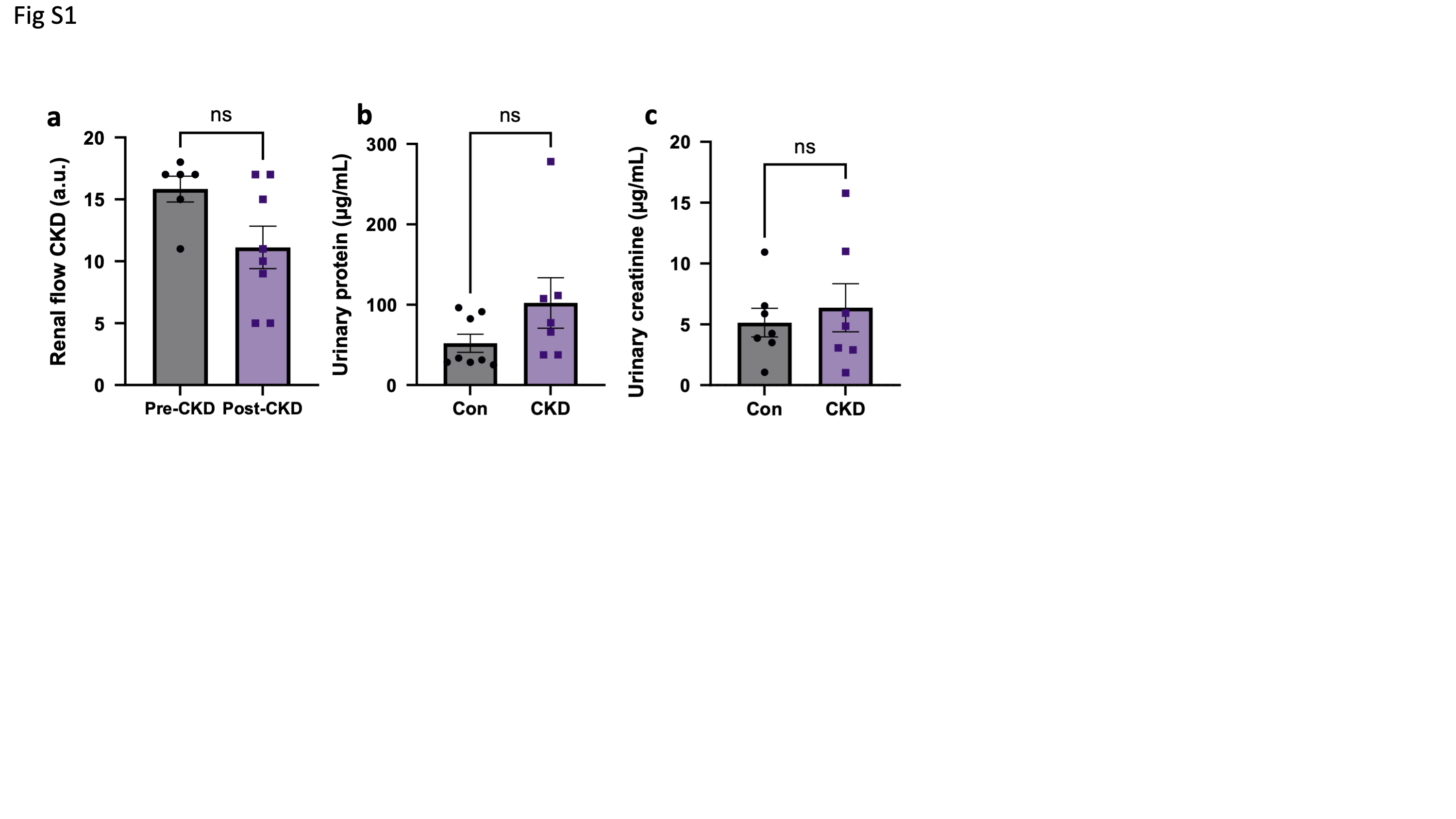

Supplement: Supplementary file 3 — Supplementary file3 (TIFF 14826 KB) [file 395_2024_1085_MOESM3_ESM.tiff]

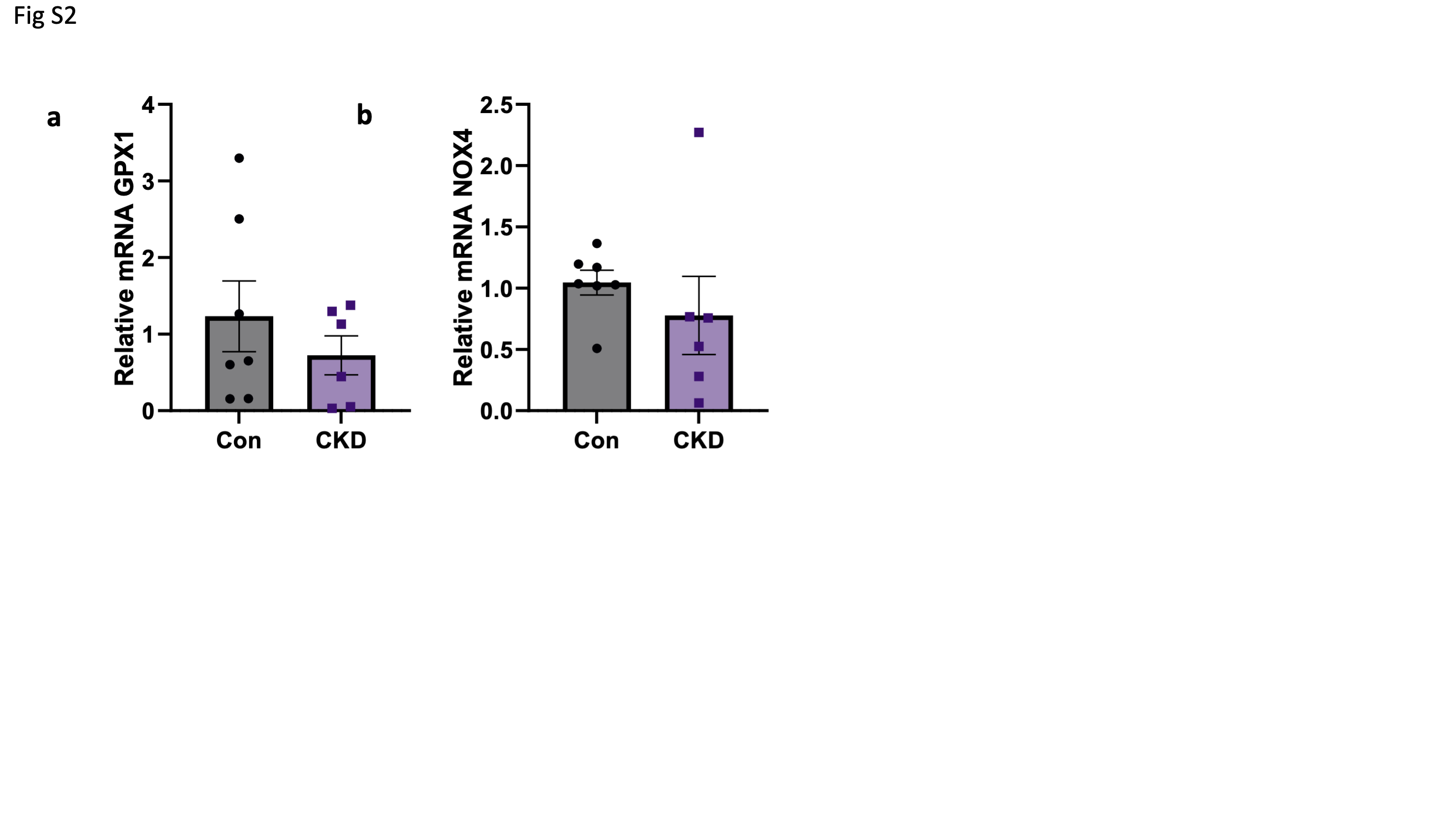

Supplement: Supplementary file 4 — Supplementary file4 (TIFF 14826 KB) [file 395_2024_1085_MOESM4_ESM.tiff]
